# Assessing the impact of physical exercise on cognitive function in older medical patients during acute hospitalization: Secondary analysis of a randomized trial

**DOI:** 10.1371/journal.pmed.1002852

**Published:** 2019-07-05

**Authors:** Mikel L. Sáez de Asteasu, Nicolás Martínez-Velilla, Fabricio Zambom-Ferraresi, Álvaro Casas-Herrero, Eduardo L. Cadore, Arkaitz Galbete, Mikel Izquierdo

**Affiliations:** 1 Navarrabiomed, Complejo Hospitalario de Navarra-Universidad Pública de Navarra, IDISNA, Pamplona, Navarra, Spain; 2 CIBER of Frailty and Healthy Aging, Instituto de Salud Carlos III, Madrid, Spain; 3 Federal University of the Rio Grande of Sul, Porto Alegre, Brazil; University of Cambridge, UNITED KINGDOM

## Abstract

**Background:**

Acute illness requiring hospitalization frequently is a sentinel event leading to long-term disability in older people. Prolonged bed rest increases the risk of developing cognitive impairment and dementia in acutely hospitalized older adults. Exercise protocols applied during acute hospitalization can prevent functional decline in older patients, but exercise benefits on specific cognitive domains have not been previously investigated. We aimed to assess the effects of a multicomponent exercise intervention for cognitive function in older adults during acute hospitalization.

**Methods and findings:**

We performed a secondary analysis of a single-blind randomized clinical trial (RCT) conducted from February 1, 2015, to August 30, 2017 in an Acute Care of the Elderly (ACE) unit in a tertiary public hospital in Navarre (Spain). 370 hospitalized patients (aged ≥75 years) were randomly allocated to an exercise intervention (n = 185) or a control (n = 185) group (usual care). The intervention consisted of a multicomponent exercise training program performed during 5–7 consecutive days (2 sessions/day). The usual care group received habitual hospital care, which included physical rehabilitation when needed. The main outcomes were change in executive function from baseline to discharge, assessed with the dual-task (i.e., verbal and arithmetic) Gait Velocity Test (GVT) and the Trail Making Test Part A (TMT-A). Changes in the Mini Mental State Examination (MMSE) test and verbal fluency ability were also measured after the intervention period. The physical exercise program provided significant benefits over usual care. At discharge, the exercise group showed a mean increase of 0.1 m/s (95% confidence interval [CI], 0.07, 0.13; *p* < 0.001) in the verbal GVT and 0.1 m/s (95% CI, 0.08, 0.13; *p* < 0.001) in the arithmetic GVT over usual care group. There was an apparent improvement in the intervention group also in the TMT-A score (−31.1 seconds; 95% CI, −49.5, −12.7 versus −3.13 seconds; 95% CI, −16.3, 10.2 in the control group; *p* < 0.001) and the MMSE score (2.10 points; 95% CI, 1.75, 2.46 versus 0.27 points; 95% CI, −0.08, 0.63; *p* < 0.001). Significant benefits were also observed in the exercise group for the verbal fluency test (mean 2.16 words; 95% CI, 1.56, 2.74; *p* < 0.001) over the usual care group. The main limitations of the study were patients’ difficulty in completing all the tasks at both hospital admission and discharge (e.g., 25% of older patients were unable to complete the arithmetic GVT, and 47% could not complete the TMT-A), and only old patients with relatively good functional capacity at preadmission (i.e., Barthel Index score ≥60 points) were included in the study.

**Conclusions:**

An individualized, multicomponent exercise training program may be an effective therapy for improving cognitive function (i.e., executive function and verbal fluency domains) in very old patients during acute hospitalization. These findings support the need for a shift from the traditional (bedrest-based) hospitalization to one that recognizes the important role of maintaining functional capacity and cognitive function in older adults, key components of intrinsic capacity.

**Trial registration:**

ClinicalTrials.gov Identifier: NCT02300896.

## Abstract 2

Mikel Izquierdo and team investigate a multicomponent exercise training program versus usual care on cognitive function among older adults during acute admission to a medical ward.

## Author summary

**Why was this study done?:**

- Acute illness requiring hospitalization is often a sentinel event leading to disability.
- Acute care hospitalization increases the likelihood of developing cognitive decline in older adults.
- Physical exercise has shown to have beneficial effects on cognition both in cognitively healthy older adults and in older adults with cognitive impairment and dementia.

**What did the researchers do and find?:**

- This study included 370 hospitalized older adults that were allocated to a physical exercise intervention group or hospital usual care group.
- An individualized multicomponent exercise intervention (i.e., progressive resistance training, balance, and gait retraining) was programmed in two daily sessions 5–7 consecutive days during hospitalization.
- The exercise program provided benefits over usual care on cognitive function in older adults.

**What do these findings mean?:**

- Physical exercise may be an effective therapy for improving cognitive function during hospitalization.
- Only old patients with relatively good functional capacity before admission were included; thus, the generalizability of the findings is limited.
- These finding support the need for a shift from the traditional bedrest hospitalization to one that recognizes the important role of maintaining functional capacity and cognitive function in older adults.

## Introduction

The provision of inpatient acute care for frail older adults has become a crucial clinical issue in our aging societies [1–3]. Acute medical illnesses and subsequent hospitalization are major events leading to disability in older people [4–6]. In addition to functional decline, acute care hospitalization increases the likelihood of developing cognitive impairment in old patients [7]. Indeed, cognitive impairment is highly prevalent in this patient group and is independently associated with multiple adverse outcomes, including functional decline, increased length of hospital stays, institutionalization, and mortality [8].

Many of the age-associated processes leading to frailty in older adults are also possibly responsible for brain aging, consecutive cognitive decline, and development of Alzheimer’s disease [9]. Accordingly, frail older people are likely to be at high risk of cognitive impairment, and vice versa [9,10]. The increasing interest in the association between frailty and cognitive impairment in hospitalized older adults [11] is driving the development of innovative interventions for the prevention and management of both conditions.

Exercise and early rehabilitation protocols applied during acute hospitalization can prevent functional decline in older patients [12] and are associated with a reduced length of stay and lower costs [13]. The exercise benefits on cognitive function are not entirely clear, but previous studies support that multicomponent exercise training seems to have the most positive effects on cognition in older adults [14,15]. To the best of our knowledge, the benefits of a multicomponent exercise intervention consisting of resistance (power), balance, and gait-retraining exercises to attenuate cognitive impairment in acutely hospitalized older adults have not been previously investigated.

The present study is in line with the long trajectory of research that has explored new possibilities to avoid dangers of prolonged bedrest [16]. Physical exercise has shown to have beneficial effects on cognition both in cognitively healthy older adults [14,17] and in older adults with cognitive impairment or dementia [18,19]. Thus, the main purpose of our study was to assess the effects of a multicomponent exercise intervention for cognitive function in older adults during acute hospitalization. Considering the strong link between physical exercise and cognition, our hypothesis was that multicomponent exercise intervention would maintain or even improve cognitive function compared to usual care in these patients.

## Methods

### Design

The study is a secondary analysis of a randomized clinical trial (RCT) (NCT02300896) [12,20]. This study is reported as per the Consolidated Standards of Reporting Trials (CONSORT) guideline (S1 CONSORT Checklist). Differing with the previous analysis, in this study, specific cognitive domains described in the study protocol [20] (i.e., executive function, verbal fluency ability) were analyzed, and other cognitive tasks such as dual tasks were included. It was conducted in the Acute Care of the Elderly (ACE) unit of the Department of Geriatrics in a tertiary public hospital (Complejo Hospitalario de Navarra, Spain). This department has 35 allocated beds, and its staff is composed of 8 geriatricians (distributed in the ACE unit, orthogeriatrics, and outpatient consultations). Admissions in the ACE unit derive mainly from the Accident and Emergency Department, with heart failure and pulmonary and infectious diseases being the main causes of admissions.

Acutely hospitalized patients who met inclusion criteria were randomly assigned to the intervention or control (usual care) group within the first 48 hours of admission. Usual care is offered to patients by the geriatricians of our department and consists of standard physiotherapy focused on walking exercises for restoring the functionality conditioned by potentially reversible pathologies. A formal exercise prescription was not provided at study entry, and patients were instructed to continue with the current activity practices through the duration of the study. The study followed the principles of the Declaration of Helsinki and was approved by the Complejo Hospitalario de Navarra Research Ethics Committee. All patients or their legal representatives provided written consent.

### Participants and randomization

All of the patients admitted to the ACE unit were evaluated by geriatricians. We focused on a particularly vulnerable population, but also one with a level of functional and cognitive capacity high enough to allow them to perform the physical exercise protocol. A trained research assistant conducted a screening interview to determine whether potentially eligible patients met the following inclusion criteria: age ≥75 years, Barthel Index score ≥60 points, and able to ambulate (with/without assistance) and to communicate and collaborate with the research team. Exclusion criteria included expected length of stay <6 days, very severe cognitive decline (i.e., Global Deterioration Scale score = 7), terminal illness, uncontrolled arrhythmias, acute pulmonary embolism and myocardial infarction, or extremity bone fracture in the past 3 months.

After the baseline assessment was performed, participants were randomly assigned following a 1:1 ratio without restrictions. The simple randomization sequence was generated by a statistician not involve in the RCT using an online system (www.randomizer.org) to allocate 185 patients in the exercise group (intervention group) and 185 patients in the usual care group (control group). Assessment staff was blinded to the main study design and group allocation. It was not possible to blind the participants, so they were explicitly informed and reminded not to discuss their randomization assignment with the assessment staff.

### Intervention

The usual care group received habitual hospital care, which included physical rehabilitation when needed. For the intervention group, exercise training was programmed in two daily sessions (morning and evening) of 20 minutes duration during 5–7 consecutive days (including weekends) supervised by a qualified fitness specialist. Adherence to the exercise intervention program was recorded in a daily register. A session was considered completed when ≥90% of the programmed exercises were successfully undertaken.

Each session was performed in a room equipped ad hoc in the ACE unit. Exercises were adapted from the “Vivifrail” multicomponent physical exercise program to prevent weakness and falls [21]. The morning sessions included individualized progressive resistance, balance, and walking-training exercises and were supervised by a physiotherapist (M.L.S.de.A) or a researcher (F.Z.F) with a PhD background in exercise physiology. The resistance exercises were tailored to the individual’s functional capacity using variable resistance training machines (Matrix; Johnson Health Tech, Ibérica, S.L., Torrejón de Ardoz, Spain and Exercycle S.L.; BHGroup, Vitoria, Spain) aiming at 2–3 sets of 8–10 repetitions with a load equivalent to 30%–60% of the estimated one-repetition maximum (1RM). Participants performed three exercises involving mainly lower-limb muscles (squats rising from a chair, leg press, and bilateral knee extension) and one involving the upper-body musculature (seated bench “chest” press). They were instructed to perform the exercises at a high speed to optimize muscle power output, and care was taken to ensure proper exercise execution. Balance and gait-retraining exercises gradually progressed in difficulty and included the following: semitandem foot standing, line walking, stepping practice, walking with small obstacles, proprioceptive exercises on unstable surfaces (foam pad sequence), altering the base of support, and weight transfer from one leg to the other. The evening session consisted of functional unsupervised exercises using light loads (0.5–1 kg anklets and hand-grip ball), such as knee extension/flexion, hip abduction, and daily walking in the corridor of the ACE unit with a duration based on the clinical physical exercise guide “Vivifrail” [21].

When the clinician in charge of the patient considered that the hemodynamic situation was acceptable and the patient could collaborate, the following endpoints were assessed, and the intervention was started. Endpoints were also assessed on the day of discharge.

### Endpoints

The primary endpoint was change in executive function during hospitalization (i.e., from admission to discharge) as assessed with the 6-meter dual-task Gait Velocity Test (GVT). Secondary endpoints were the Mini Mental State Examination (MMSE) test, the Trail Making Test Part A (TMT-A), and the verbal fluency test. The MMSE endpoint was included in the main analysis of the RCT [12].

### 6-meter dual-task GVT

Patients were instructed to walk at their self-selected usual pace on a smooth, horizontal walkway. Two different dual-task gait tests were performed, the arithmetic GVT and the verbal GVT, in which gait velocity was measured while the patients counted backward aloud from 100 down to 1 or named animals aloud, respectively [22,23]. The cognitive score was measured by counting the number of animals named (verbal dual task) or by counting the numbers that were stated (arithmetic dual task) and the errors in each task.

### MMSE

The MMSE test [24] is the most utilized screening instrument of cognitive decline [25]. The instrument assesses domains of orientation, memory, attention, language, and visuospatial ability. The MMSE is scored out of 30 points, with scores ≤23 points indicative of likely cognitive impairment.

### TMT-A

The TMT-A is used as an indicator of visual scanning, graphomotor speed, and executive function. The patients were asked to connect randomly arranged circles containing numbers from 1 to 25 following the number sequence and to do it as quickly as possible [26].

### Verbal fluency test

The patient had to say as many words as possible starting with the letter F in one minute [27].

### Statistical analysis

Analyses were performed by “intention-to-treat” principles. After analyzing missing-data patients in both groups and comparing them with the non-missing–data patients, a missing at random (MAR) mechanism was assumed.

Between-group comparisons of continuous variables were conducted using linear mixed models. Time was treated as a categorical variable. The models included group, time, and group by time interaction as fixed effects and participants as random effect. For each group, data are expressed as change from baseline (admission) to discharge, determined by the time coefficients (95% confidence interval [CI]) of the model. The conclusions about effectiveness of exercise intervention were based on between-group comparisons of change in cognitive function from baseline (beginning of the intervention) to hospital discharge, as assessed with the MMSE, dual-task GVT (including both verbal and arithmetic task conditions), TMT-A, and verbal fluency test and determined by the time by group interaction coefficients of the model. Between-group comparisons of errors during the dual-task GVTs were analyzed using the Poisson mixed model because of the asymmetric distribution of the endpoint.

Using the χ^2^ test for linear trend, we also compared the proportion of patients in each group showing an improvement, no change, or worsening at discharge at compared with baseline on the dual-task GVTs.

Normality of data was checked graphically and through the Kolmogorov–Smirnov test. The residuals were also checked graphically, and no noticeable deviation from normality was observed. All comparisons were two-sided, with a significance level of 0.05. Statistical analysis was carried out using IBM-SPSS v20 software (SPSS Inc., Chicago, IL, USA).

## Results

**Fig 1:**
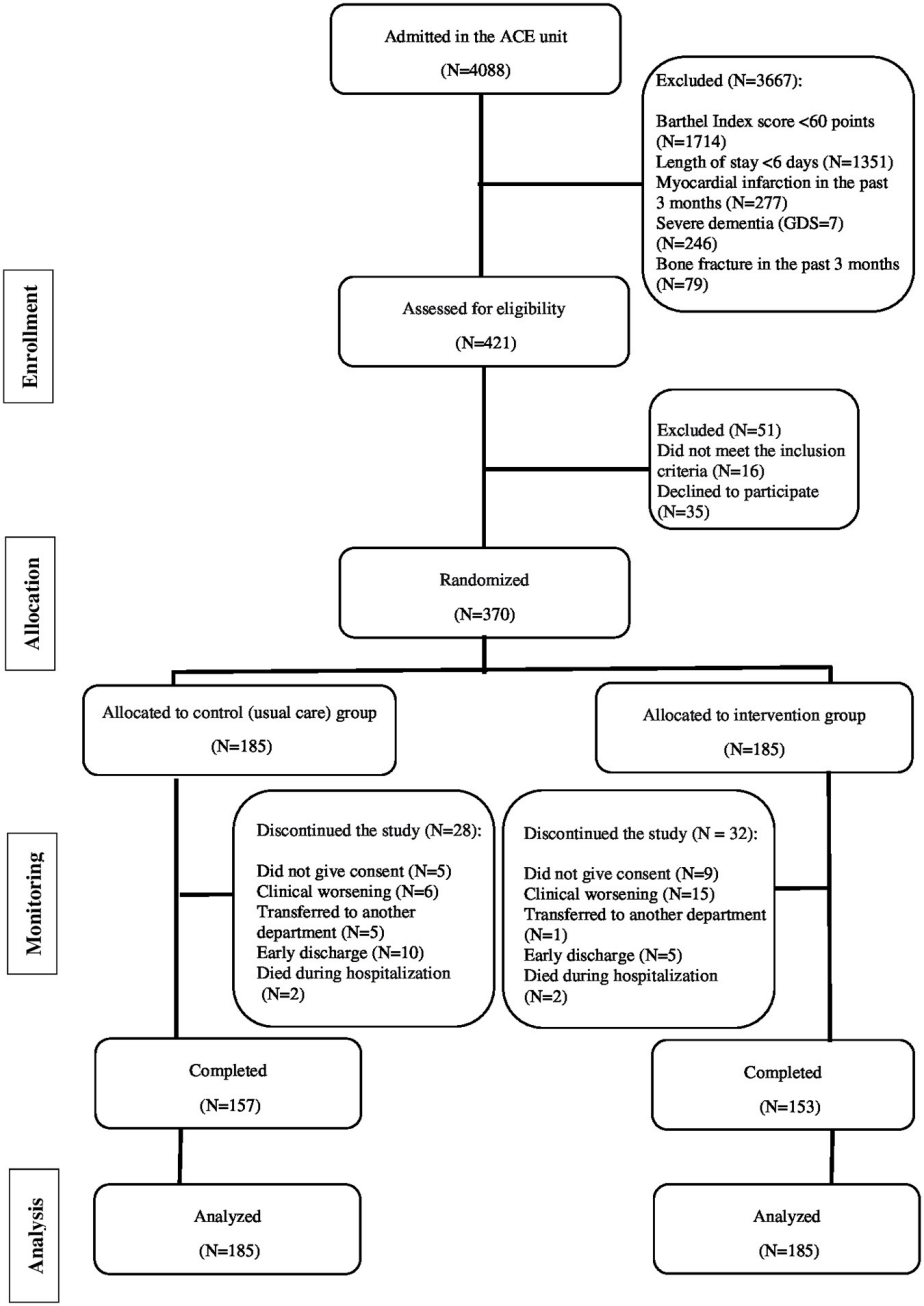
Study flow diagram. ACE, Acute Care of the Elderly; GDS, Yesavage Geriatric Depression Scale.

The study flow diagram is shown in Fig 1. No significant differences were found between groups at baseline for demographic and clinical characteristics for study endpoints (Table 1). Of the 370 patients included in the analyses, 209 were women (56.5%); the mean age was 87.3 (4.9) years (range 75–101 years), with 130 patients (35.1%) being nonagenarians). The median length of hospital stay was 8 days in both groups (interquartile range [IQR], 4 and 4 days, respectively). The mean number of intervention days for each patient was 5.3 ± 0.5 days, with most training days being consecutive (97%). The number of completed morning and evening sessions per patient averaged 5 ± 1 and 4 ± 1, respectively. Mean adherence to the intervention was 97% (95% CI, 95.67, 98.36) for the morning sessions (i.e., 806 successfully completed sessions of 841 total possible sessions) and 85% (95% CI, 79.70, 89.40) in the evening sessions (574 of 688). No adverse effects or falls associated with the prescribed exercises were recorded, and no patient had to interrupt the intervention or had their hospital stay modified because of it.

**Table 1:** Baseline characteristics of the participants.

Variable	Control Group (n = 185)	Intervention Group (n = 185)
**Demographic data**		
Age, years	87.1 (5.2)	87.6 (4.6)
Women, N (%)	109 (59%)	100 (54%)
Body mass index, kg/m^2^	26.9 (4.9)	27.1 (4.4)
**Clinical data**		
Barthel Index, score	83 (17)	84 (17)
CIRS score, median (IQR)	12 (5)	13 (5)
MNA score, median (IQR)	24 (4)	24 (4)
1RM leg press, kg	62 (31)	57 (25)
1RM chest press, kg	25 (12)	24 (11)
1RM knee extension, kg	41 (14)	39 (13)
GDS, score	3.6 (2.9)	4.0 (2.4)
QoL (EQ-VAS), score	60 (21)	58 (22)
Delirium (CAM, %)	12%	17%
**Endpoint measures**		
Verbal GVT, m/s	0.4 (0.2)	0.4 (0.2)
Arithmetic GVT, m/s	0.4 (0.2)	0.4 (0.2)
MMSE, score	23 (4)	22 (5)
TMT-A, seconds	162.9 (97.0)	166.5 (125.4)
Verbal fluency test, score	7.2 (4.2)	6.3 (3.8)
**Admission reason, N (%)**		
Cardiovascular	67 (36)	65 (35)
Infectious	33 (18)	33 (18)
Pulmonary	20 (11)	28 (15)
Gastrointestinal	17 (9)	20 (11)
Neurological	9 (5)	9 (5)
Other	39 (21)	30 (16)

Data are mean (SD) unless otherwise stated. No statistically significant differences were found between groups (all *p* > 0.05).

**Abbreviations**: CAM, Confusion Assessment Method; CIRS, Cumulative Illness Rating Scale; EQ-VAS, visual analogue scale of the EuroQol questionnaire (EQ-5D); GDS, Yesavage Geriatric Depression Scale; GVT, Gait Velocity Test; IQR, interquartile range; MMSE, Mini Mental State Examination; MNA, Mini-nutritional Assessment; QoL, quality of life; SPPB, Short Physical Performance Battery; TMT-A, Trail Making Test Part A; 1RM, one-repetition maximum.

**Fig 2:**
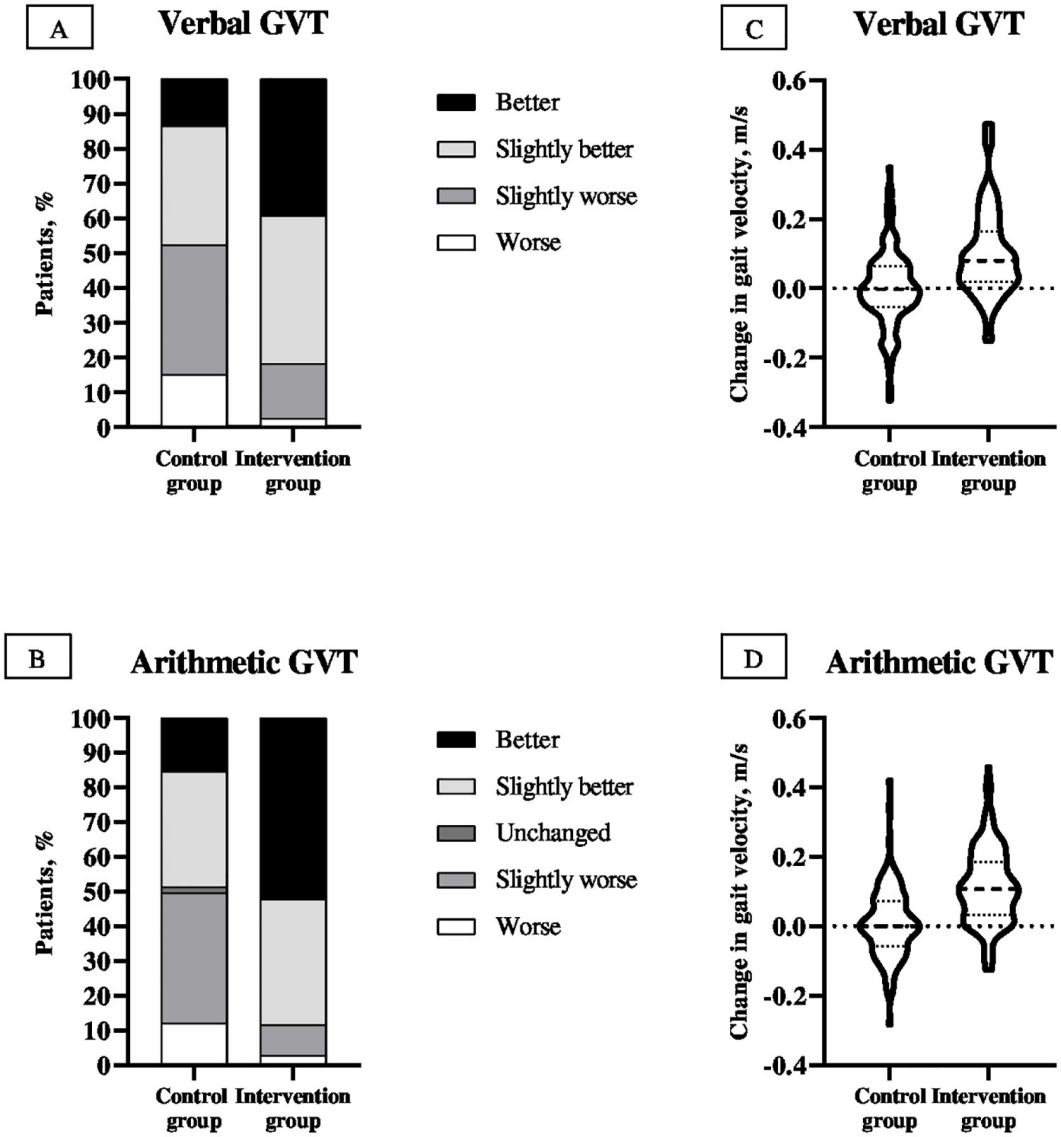
Changes from baseline to discharge (A and B) and within-group punctuation change distribution (C and D). Dual-task GVT changes: “better” indicates an improvement of more than 0.1 m/s, “slightly better” indicates an improvement between 0.001 and 0.1 m/s, “unchanged” indicates no difference, “slightly worse” indicates a decline between 0.001 and 0.1 m/s, and “worse” indicates a decline of more than 0.1 m/s. The proportion of patients showing overall improvement and worsening in the dual-task GVTs was significantly higher and lower, respectively, in the intervention than in the control group (all *p* < 0.001 with χ^2^ test). In the violin plots, the horizontal dotted lines indicate Q1 and Q3, and the horizontal dashed line within the violin, the median. GVT, Gait Velocity Test; Q1, First Quartile; Q3, Third Quartile.

The primary analysis showed that the physical exercise seems to provide a significant benefit over usual care. Differences between the treatment groups revealed a significant intervention effect for both dual-task GVTs. The percentage distribution of patients with improvements on the verbal GVT (47.6% versus 81.7%) or arithmetic GVT (48.7% versus 88.5%) from admission to discharge significantly differed between the two groups, indicating a beneficial exercise intervention effect for both endpoints (all *p* < 0.001 with χ^2^ test, Fig 2A and 2B). At discharge, the exercise group showed an increase of 0.1 m/s (95% CI, 0.07, 0.13 m/s; *p* < 0.001) on the verbal GVT and 0.1 m/s (95% CI, 0.08, 0.13 m/s; *p* < 0.001) on the arithmetic GVT over the usual care group (Table 2, Fig 2C and 2D). Furthermore, significant enhancements were found in the intervention group in the errors made during the arithmetic GVT (0.48 errors; 95% CI, 0.34, 0.67 words) over the control group (*p* < 0.001, Table 2).

**Table 2:** Results of study endpoints by group.

Endpoints	Control Group	Exercise Group	Between-Group Difference (95% CI)	*p*-Value Between Groups
**Verbal GVT**				
Velocity, m/s	0.002 (−0.018, 0.022)	0.10 (0.08, 0.12)	0.10 (0.07, 0.13)	<0.001
Correct answers, score	0.01 (−0.36, 0.38)	0.41 (0.04, 0.79)	0.41 (−0.12, 0.93)	0.133
Errors, score*	2.03 (0.64, 7.61)	0.32 (0.016, 2.41)	0.16 (0.01, 1.65)	0.157
**Arithmetic GVT**				
Velocity, m/s	0.009 (−0.009, 0.029)	0.11 (0.10, 0.13)	0.10 (0.08, 0.13)	<0.001
Correct answers, score	0.12 (−0.57, 0.81)	0.18 (−0.52, 0.88)	0.06 (−0.92, 1.05)	0.901
Errors, score*	1.16 (0.92, 1.45)	0.55 (0.42, 0.69)	0.48 (0.34, 0.67)	<0.001
**MMSE, score**	0.27 (−0.08, 0.63)	2.10 (1.75, 2.46)	1.83 (1.32, 2.33)	<0.001
**TMT-A, seconds**	−3.13 (−16.3, 10.2)	−34.2 (−47.1, −21.3)	−31.1 (−49.5, −12.7)	<0.001
**Verbal fluency test**				
Correct answers, score	−0.30 (−0.72, 0.12)	1.85 (1.44, 2.27)	2.16 (1.56, 2.74)	<0.001
Errors, score*	1.11 (0.75, 1.63)	0.66 (0.43, 0.99)	0.58 (0.33, 1.05)	0.076

Data in each group are expressed as change from baseline (admission) to discharge (mean and 95% CI). A total of 247 patients (79% of 310 patients who completed the intervention) were able to perform the verbal GVT at admission and discharge; 231 patients (75%) the arithmetic GVT; 292 older adults (94%) the MMSE test; 162 patients (53%) the TMT-A; and 289 patients (93%) the verbal fluency test.

*Poisson mixed model. Effects are rate ratios: within-group effects are time coefficients in the model, and between-group effects are group-time interaction coefficients.

**Abbreviations**: CI, confidence interval; GVT, Gait Velocity Test; MMSE, Mini Mental State Examination; TMT-A, Trail Making Test Part A.

**Fig 3:**
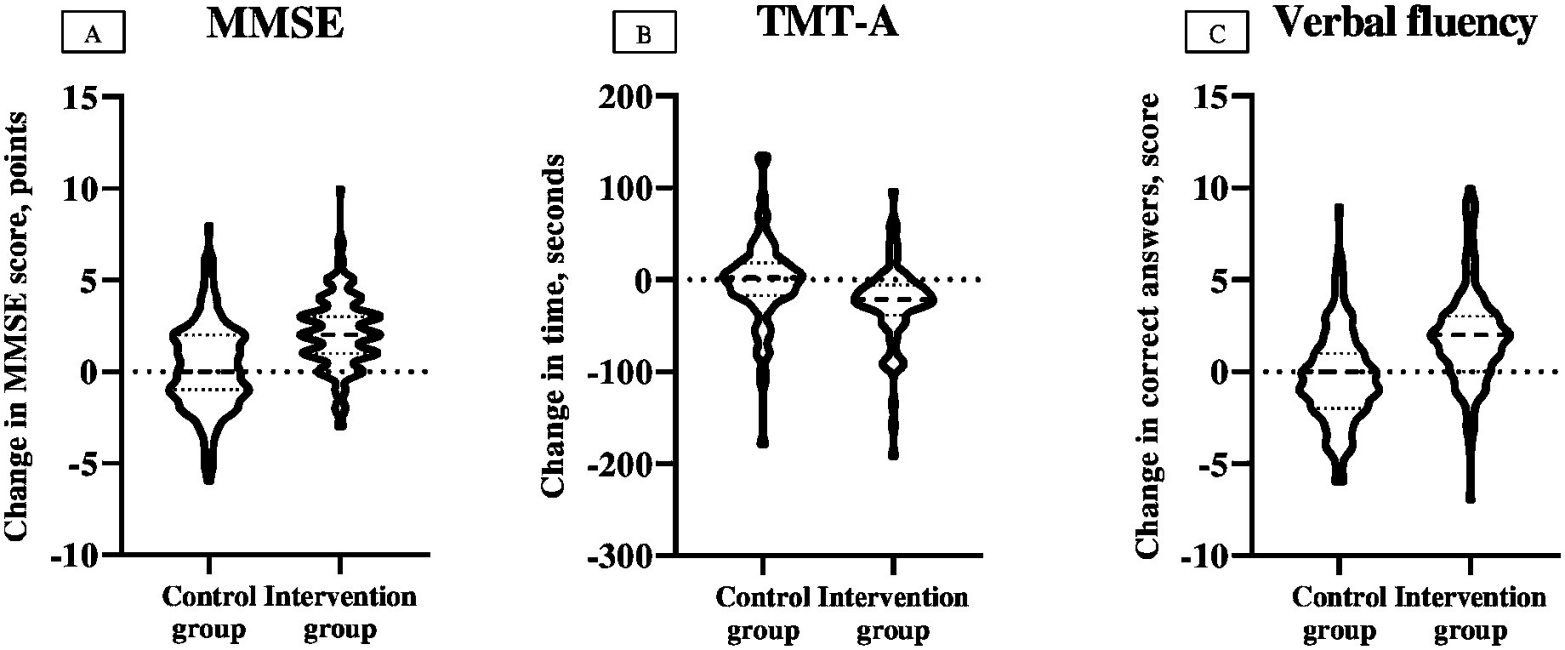
Changes in within-group punctuation in the MMSE test, TMT-A, and verbal fluency test. In the violin plots, the horizontal dotted lines indicate Q1 and Q3, and the horizontal dashed line within the violin, median. MMSE, Mini Mental State Examination; Q1, First Quartile; Q3, Third Quartile; TMT-A, Trail Making Test Part A.

Considering the global cognitive function, the intervention group showed improvements at discharge in the MMSE test of 2.10 points (95% CI, 1.75, 2.46 points), whereas no such trend was found in the control group (0.27 points; 95% CI, −0.08, 0.63 points) (*p* < 0.001) (Table 2 and Fig 3A).

For the executive function, the exercise group showed an improvement in the TMT-A, reducing the time to complete the task by 31.1 seconds at discharge (95% CI, −49.5 to −12.7 seconds; *p* < 0.001) over the control group (Table 2 and Fig 3B).

Finally, acute hospitalization per se led to a significant impairment in patient verbal fluency ability (i.e., mean change from baseline to discharge of −0.30 words [95% CI, −0.72, 0.12 words]), whereas the exercise intervention improved this cognitive domain (1.85 words; 95% CI, 1.44, 2.27 words) (*p* < 0.001) (Table 2 and Fig 3C).

## Discussion

This secondary analysis of the RCT suggests that an individualized exercise intervention, delivered over a mean of 5 days, may provide benefits over usual care in acutely hospitalized older adults and may help reverse the cognitive impairment often associated with this patient group. To our knowledge, this is the first study to point towards the beneficial effects of a multicomponent intervention, including low-intensity resistance training exercises on specific cognitive domains such as executive function and verbal fluency, in hospitalized patients of advanced age.

Older patients admitted to the hospital are at risk of experiencing negative consequences following hospitalization, including functional decline and, frequently, long-term disability [5,6]. Research has suggested that hospitalization in older adults per se is associated not only with functional adverse outcomes but also with the development of cognitive decline and an increased risk of dementia [7]. Moreover, cognitively impaired older patients are at even greater risk of hazards of hospital stay as compared to patients with no cognitive decline [28]. Our findings reveal that more than one-half of the control group showed worsened gait performance in both the dual-task GVTs at discharge, whereas the exercise intervention reversed this trend. We also observed an improvement in the verbal fluency ability after the exercise intervention, with the opposite response found in the usual care group. Surprisingly, short-term hospitalization did not impact dramatically on some cognitive tasks, such as the MMSE and TMT-A, although significant differences were observed between groups at discharge. The poor health status of the hospitalized old patients upon admission and the comprehensive and multidisciplinary protocols already established in the ACE unit could influence the preservation of some cognitive domains.

Acute hospital admissions play a key role in the disabling process in older adults, and physical exercise seems to be an effective therapy to prevent nosocomial disability, which is usually linked to poor mobility during hospitalization [29]. Recent evidence has demonstrated that specific in-hospital exercises could provide significant benefits over usual care and could help to reverse the functional decline associated with acute hospitalization in older adults [12]. Although potential benefits of physical exercise on functional capacity are well established, the effects of tailored multicomponent exercise intervention on specific cognitive domains including executive function and verbal fluency are not clear in acutely hospitalized older patients. In agreement with previous studies [14,15], our findings support that multicomponent exercise training may produce the most positive effects on cognitive function in older adults. The inclusion of progressive low-intensity resistance training as a component of the exercise training protocol could be the reason for cognitive gains in the intervention group in specific executive tasks (i.e., both dual-task GVTs and TMT-A). An emerging theory to explain these cognitive benefits is that resistance training increases the production of several growth factors, such as brain-derived neurotrophic factor and insulin-like growth factor 1 [30]. Previous evidence has suggested that gait performance is closely related to cognitive function, in particular executive function, and impaired executive function has been associated with decreased gait velocity, increased risk of falls, and decreased performance on complex motor tasks in older adults [31,32]. Thus, our results indicate that, despite its short duration, an exercise training approach can be effective in improving the executive function (measured by dual-task GVT) during hospitalization in very old patients.

The present study is in line with the recently published World Health Organization (WHO) Clinical Consortium of Healthy Aging, which highlights the importance of maintaining individuals’ intrinsic capacity for the preservation of autonomy and independence in essential everyday activities [33]. Our findings suggest that a multicomponent exercise program with special emphasis on muscle-power training may help mitigate the trajectory towards frailty or disability in acutely hospitalized older adults and appeared to improve cognitive function, a key component of intrinsic capacity. These data suggest that, in accordance with the WHO framework, exercise prescription should be considered as part of frontline treatment to prevent hospital-acquired iatrogenic disability. Future RCTs should also consider the inclusion of multidomain interventions, in which exercise training is combined with other treatments such as cognitive training and social enrichment, in this population to optimize cognitive performance and prevent cognitive impairment.

Our study has several strengths. We focused on a particularly vulnerable population of advanced age (overall mean 87.3 years; range 75–101 years, with 130 patients [35.1%] being nonagenarians) to develop an innovative exercise intervention of a few days (i.e., 5 ± 1 and 4 ± 1 morning and evening sessions, respectively) in acute settings. Also, patients with multiple comorbidities (mean [SD] of 9 [6] comorbidities) and mild dementia/cognitive impairment were included in the RCT (routinely excluded from exercise studies). Considering the exercise training protocol, a daily individualized adjustment of loads was performed to optimize exercise benefits and prevent iatrogenic nosocomial disability. In order to minimize potential bias, the researchers were unaware of patient test scores at admission when retesting at discharge. Our study, nevertheless, has some limitations, including patients’ difficulty in completing all the tasks at both hospital admission and discharge. Notably, 25% of the older patients were unable to perform the arithmetic GVT, mainly because they did not receive primary education, and 47% of the participants could not complete the TMT-A because of visual impairment. Thus, the missing data were not possible to collect because of the characteristics of the study population (octogenarians and nonagenarians with multiple geriatric syndromes). However, the data missingness was not differential between the intervention and control group, and it seems not to affect to the between-group differences. The generalizability of our results is limited due to the inclusion of a selected population with relatively good functional capacity at preadmission (i.e., Barthel Index score ≥60 points), excluding those older adults with severe dementia, with unstable hemodynamic condition, or not able to walk at admission. Physical exercise during acute hospitalization may be beneficial for avoiding bedrest complications, but further research is needed in other hospitalized older medical populations, and it would be interesting to replicate our findings in a multicenter study. Additionally, it was not possible to blind physical therapists in charge of supervising the intervention, patients, or families to the group assignment of a patient given the nature of the intervention. Finally, delirium data were not included in this secondary analysis because they were added in the main analysis of the RCT [12].

Our findings point to several future directions for research. In general, physical exercise intervention seems to provide benefits on cognition over usual care in acutely hospitalized older adults. Future studies should analyze the individual response of participants to usual care (control group) and to physical exercise (intervention group) for cognitive function to prescribe more individualized treatments and optimize the results. Additionally, the role of exercise training on cognition at follow-up (i.e., post-discharge) needs to be further explored in trials.

In this study, we evaluated the effects of an individualized, multicomponent exercise program on cognitive function in hospitalized older adults. The physical exercise intervention appears to be an effective therapy for improving cognitive function (i.e., executive function and verbal fluency domains) in very old patients during acute hospitalization. These findings support the need for a shift from the traditional (bedrest-based) hospitalization to one that recognizes the important role of maintaining functional capacity and cognitive function in older adults, key components of intrinsic capacity.

## Supporting information

S1 CONSORT Checklist CONSORT 2010 checklist. (DOC)

S1 Data (XLSX)

## Abbreviations

1RM: one-repetition maximum
ACE: Acute Care of the Elderly
CAM: Confusion Assessment Method
CI: Confidence Interval
CIRS: Cumulative Illness Rating Scale
CONSORT: Consolidated Standards of Reporting Trials
EQ-VAS: visual analogue scale of the EuroQol questionnaire (EQ-5D)
GDS: Yesavage Geriatric Depression Scale
GVT: Gait Velocity Test
IQR: interquartile range
MAR: missing at random
MMSE: Mini Mental State Examination
MNA: Mini-nutritional Assessment
Q1: First Quartile
Q3: Third Quartile
QoL: Quality of Life
RCT: randomized clinical trial
SPPB: Short Physical Performance Battery
TMT-A: Trail Making Test Part A
