# Supplementary material for: Assessing the impact of physical exercise on cognitive function in older medical patients during acute hospitalization: Secondary analysis of a randomized trial
Source: PLoS Med. 2019 Jul 5;16(7):e1002852. doi: 10.1371/journal.pmed.1002852 (PMC6611563; doi:10.1371/journal.pmed.1002852)
Supplement: S1 CONSORT Checklist — (DOC) [file pmed.1002852.s001.doc]

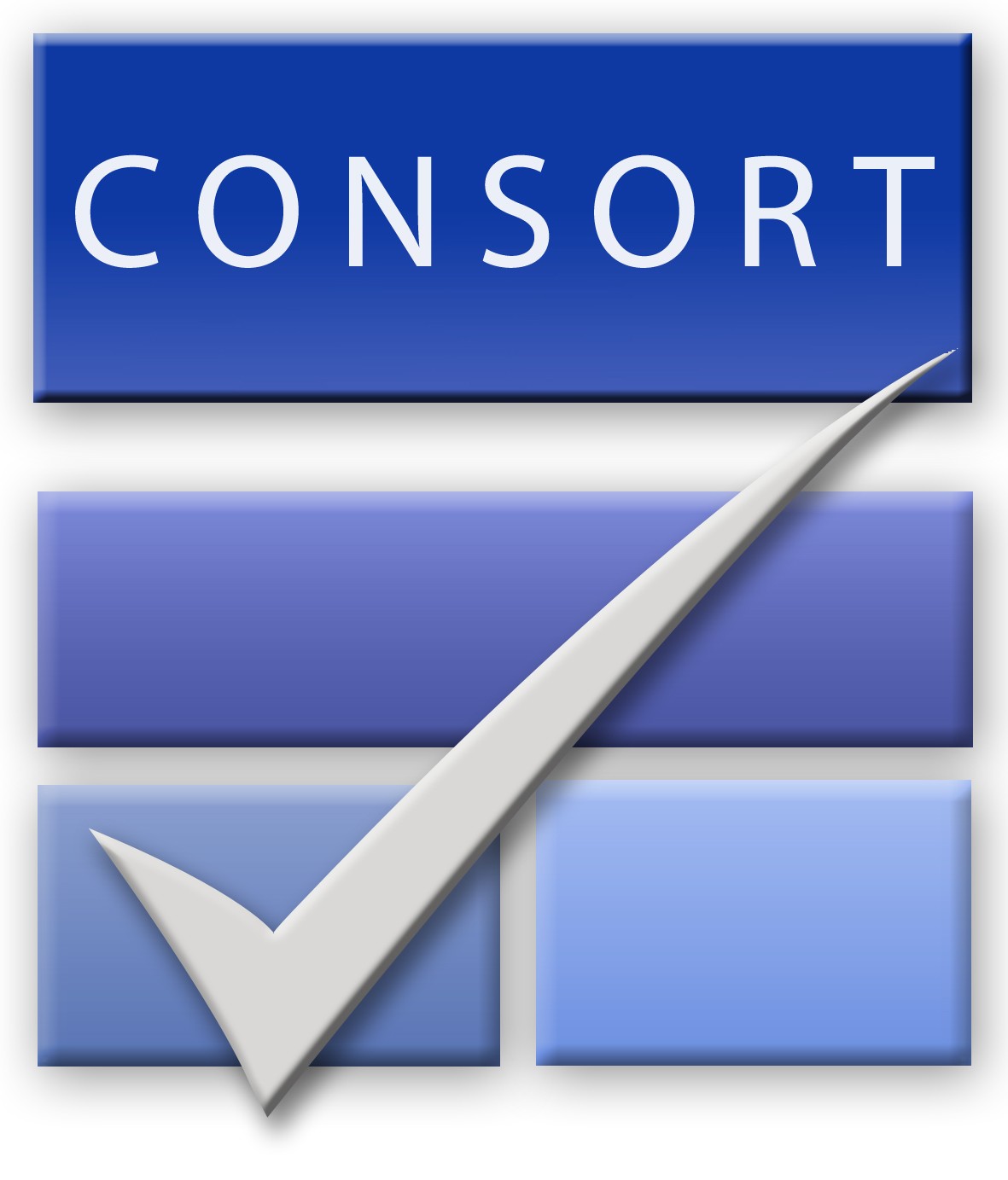
CONSORT 2010 checklist of information to include when reporting a randomised trial*

| Section/Topic | Item No | Checklist item | Reported on page No |
| --- | --- | --- | --- |
| Title and abstract | | | |
|  | 1a | Identification as a randomised trial in the title | Page 1 |
|  | 1b | Structured summary of trial design, methods, results, and conclusions (for specific guidance see CONSORT for abstracts) | Page 2 |
| Introduction | | | |
| Background and objectives | 2a | Scientific background and explanation of rationale | Introduction page 1 |
|  | 2b | Specific objectives or hypotheses | Introduction Paragraph 4 |
| Methods | | | |
| Trial design | 3a | Description of trial design (such as parallel, factorial) including allocation ratio | Methods  Paragraph 1 |
|  | 3b | Important changes to methods after trial commencement (such as eligibility criteria), with reasons | Methods  Paragraph 2 |
| Participants | 4a | Eligibility criteria for participants | Methods  Paragraph 3 |
|  | 4b | Settings and locations where the data were collected | Methods  Paragraph 4 |
| Interventions | 5 | The interventions for each group with sufficient details to allow replication, including how and when they were actually administered | Methods  Paragraph 5-6 |
| Outcomes | 6a | Completely defined pre-specified primary and secondary outcome measures, including how and when they were assessed | Methods  Paragraph 8-12 |
|  | 6b | Any changes to trial outcomes after the trial commenced, with reasons | Methods  Paragraph 8-12 |
| Sample size | 7a | How sample size was determined | Methods  Paragraph 1 |
|  | 7b | When applicable, explanation of any interim analyses and stopping guidelines | Methods  Paragraph 1 |
| Randomisation: |  |  |  |
| Sequence generation | 8a | Method used to generate the random allocation sequence | Methods  Paragraph 4 |
|  | 8b | Type of randomisation; details of any restriction (such as blocking and block size) | Methods  Paragraph 4 |
| Allocation concealment mechanism | 9 | Mechanism used to implement the random allocation sequence (such as sequentially numbered containers), describing any steps taken to conceal the sequence until interventions were assigned | Methods  Paragraph 4 |
| Implementation | 10 | Who generated the random allocation sequence, who enrolled participants, and who assigned participants to interventions | Methods  Paragraph 4 |
| Blinding | 11a | If done, who was blinded after assignment to interventions (for example, participants, care providers, those assessing outcomes) and how | Methods  Paragraph 4 |
|  | 11b | If relevant, description of the similarity of interventions | Methods  Paragraph 5 |
| Statistical methods | 12a | Statistical methods used to compare groups for primary and secondary outcomes | Methods  Paragraph 13-15 |
|  | 12b | Methods for additional analyses, such as subgroup analyses and adjusted analyses | Methods  Paragraph 13-15 |
| Results | | | |
| Participant flow (a diagram is strongly recommended) | 13a | For each group, the numbers of participants who were randomly assigned, received intended treatment, and were analysed for the primary outcome | Results  Paragraph 1 |
|  | 13b | For each group, losses and exclusions after randomisation, together with reasons | Results  Paragraph 1 |
| Recruitment | 14a | Dates defining the periods of recruitment and follow-up | Results  Paragraph 1 |
|  | 14b | Why the trial ended or was stopped | Results  Paragraph 1 |
| Baseline data | 15 | A table showing baseline demographic and clinical characteristics for each group | Results  Paragraph 1 |
| Numbers analysed | 16 | For each group, number of participants (denominator) included in each analysis and whether the analysis was by original assigned groups | Results  Paragraph 1 |
| Outcomes and estimation | 17a | For each primary and secondary outcome, results for each group, and the estimated effect size and its precision (such as 95% confidence interval) | Results  Paragraph 2-6 |
|  | 17b | For binary outcomes, presentation of both absolute and relative effect sizes is recommended | Results  Paragraph 2-6 |
| Ancillary analyses | 18 | Results of any other analyses performed, including subgroup analyses and adjusted analyses, distinguishing pre-specified from exploratory | Results  Paragraph 2-6 |
| Harms | 19 | All important harms or unintended effects in each group (for specific guidance see CONSORT for harms) | Results  Paragraph 2-6 |
| Discussion | | | |
| Limitations | 20 | Trial limitations, addressing sources of potential bias, imprecision, and, if relevant, multiplicity of analyses | Discussion  Paragraph 5 |
| Generalisability | 21 | Generalisability (external validity, applicability) of the trial findings | Discussion  Paragraph 4-5 |
| Interpretation | 22 | Interpretation consistent with results, balancing benefits and harms, and considering other relevant evidence | Discussion  Paragraph 5-6 |
| Other information | | |  |
| Registration | 23 | Registration number and name of trial registry | Methods  Paragraph 1 |
| Protocol | 24 | Where the full trial protocol can be accessed, if available | Methods Paragraph 1 |
| Funding | 25 | Sources of funding and other support (such as supply of drugs), role of funders | Funding  Paragraph 1 |

*We strongly recommend reading this statement in conjunction with the CONSORT 2010 Explanation and Elaboration for important clarifications on all the items. If relevant, we also recommend reading CONSORT extensions for cluster randomised trials, non-inferiority and equivalence trials, non-pharmacological treatments, herbal interventions, and pragmatic trials. Additional extensions are forthcoming: for those and for up to date references relevant to this checklist, see [www.consort-statement.org](http://www.consort-statement.org/).
